# Cuidado de Enfermería del Primer Paciente Neonato con ECMO Central Veno-arterial más *Venting* en Perú

**DOI:** 10.47487/apcyccv.v1i2.47

**Published:** 2020-06-29

**Authors:** Gianina Alvarado-Soto, María Elena Barrios-Sarmiento, Silvia León-Lázaro

**Affiliations:** 1 Enfermera asistencial especialista en cuidados cardiovasculares pediátricos. Servicio de Cardiopediatría. Instituto Nacional Cardiovascular - INCOR EsSalud. Lima, Perú. Enfermera asistencial especialista en cuidados cardiovasculares pediátricos. Servicio de Cardiopediatría. Instituto Nacional Cardiovascular - INCOR EsSalud Lima Perú

**Keywords:** ECMO, cirugía cardíaca, cuidados de enfermería, neonato, ECMO, cardiac surgery, nursing care, newborn

## Abstract

Se reporta los cuidados de enfermería del primer paciente neonato en el Perú que usó oxigenación por membrana extracorpórea (ECMO) central veno-arterial (VA) y cánula de descompresión de aurícula izquierda (VENT). Se estandarizó el cuidado de enfermería especializado identificándose 15 diagnósticos según la taxonomía II NANDA y 3 que podrían incorporarse a ella para el cuidado del neonato en ECMO.

## Abstract (en)

We report the nursing care of the first newborn patient in Peru who used central veno-arterial (VA) extracorporeal membrane oxygenation (ECMO) - plus left atrial decompression cannula (VENT). We standardized specialized nursing care by identifying 15 diagnoses according to taxonomy II nursing diagnoses (NANDA) and 3 that could be incorporated into it for newborn care in ECMO.

## Introducción

La oxigenación con membrana extracorpórea (ECMO por sus siglas en inglés) brinda un período de descanso al corazón/pulmón dependiendo del tipo de soporte^. (^^1^ ECMO es una terapia invasiva con riesgos potenciales, por lo que existen protocolos internacionales que determinan el tipo de pacientes a quienes se les puede ofrecer dicha terapia. La Organización de Soporte Vital Extracorpóreo (ELSO por sus siglas en inglés) es la que nos brinda protocolos y directrices para su manejo adecuado. 

ECMO es una terapia que se usó inicialmente en neonatos con falla respiratoria (1975), siendo actualmente aceptada para recién nacidos, niños y adultos en falla respiratoria y(o) cardíaca potencialmente reversibles, que no responden al tratamiento médico convencional o cuando este es insuficiente y puede incidir negativamente en el pronóstico del paciente.^1^ Es aquí donde la terapia del ECMO obtiene mayor supervivencia sin incrementar secuelas; siendo para ello necesario contar con la tecnología y con un equipo multidisciplinario capacitado en su manejo.

Se presenta el primer caso de un neonato en ECMO en el Instituto Nacional Cardiovascular - INCOR EsSalud de Lima.

## Descripción del Caso

El paciente presentó al nacimiento el diagnóstico de transposición de grandes vasos (TGV), ductus arterioso (PCA) dependiente y comunicación interauricular (CIA) restrictiva. Fue tratado inicialmente con prostaglandinas en su hospital de origen y luego transferido al Instituto Nacional Cardiovascular - INCOR EsSalud donde a los 23 días de vida fue sometido a un procedimiento de Rashkind, que consiste en realizar una atrioseptostomía con balón o ampliar el foramen oval mediante cateterismo cardíaco.

Luego del procedimiento, se evidenció leve mejoría hemodinámica pero también desaturación con repercusión en la oxigenación tisular. Por ello a los 25 días de vida fue llevado a cirugía correctiva con técnica de Jatene. El tiempo de circulación extracorpórea fue de 4 horas y 33 minutos y el tiempo de clampaje de aorta, de 1 hora con 44 minutos.

**Figura 1:**
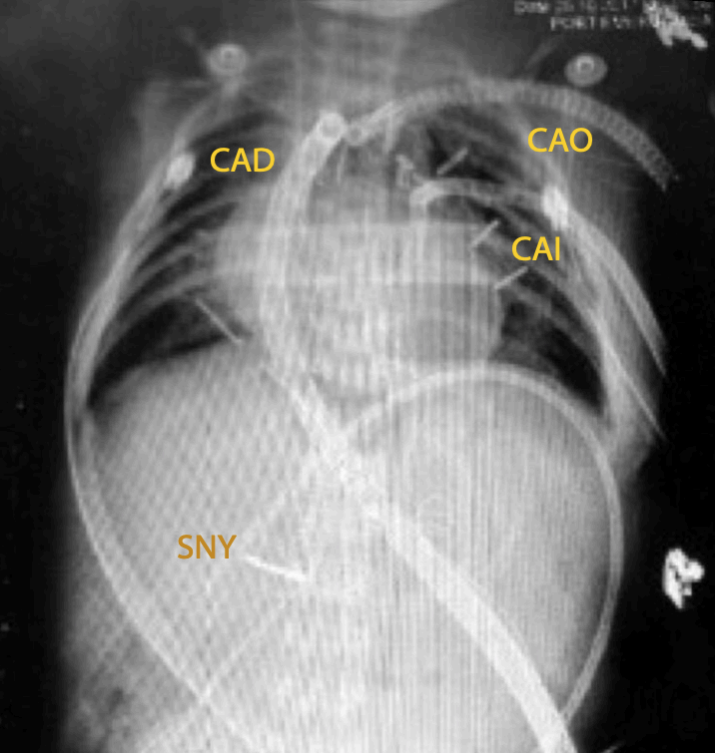
Radiografía del paciente en ECMO que permite identificar algunos elementos del sistema ECMO.

CAD: cánula de aurícula derecha Medtronic 14F; CAO: cánula aórtica Stockert N°10; CAI: cánula de descompresión de atrio izquierdo Medtronic 12F; SNY: sonda nasoyeyunal.

La evolución postoperatoria fue desfavorable. En el día postoperatorio 2 (27 días de vida), ya requería alto apoyo inotrópico (incluyendo uso de levosimendán), y lactato de 9 mmol/L. La ecocardiografía mostró dilatación de cavidades izquierdas con hipocinesia global, dilatación de seno coronario y una fracción de eyección del ventrículo izquierdo de 18%. Por ello, se decide implante de ECMO central veno-arterial más cánula de descompresión del atrio izquierdo (VENT). (Figuras 1 y 2)

**Figura 2:**
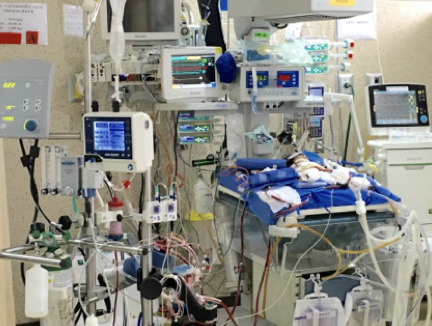
Paciente en ECMO veno-arterial con descompresión de atrio izquierdo. ECMO marca Sorin.

Durante el soporte con ECMO, se controló el tiempo de tromboplastina parcial (TTP) y el tiempo de coagulación activada (TCA) haciendo uso de la guía de infusión de heparina del paciente en ECMO, adaptada y modificada según evidencia de resultado. Al no contar con máquinas de hemofiltrado, se usó el ultrafiltrado DIDECO pediátrico. El proceso de ultrafiltrado duraba tres o cuatro horas, pero en ocasiones se realizó de forma continua, haciendo vigilancia del medio interno del paciente y de su hemodinámica. 

A los 31 días de vida, habiéndose comprobado la normalización de la función sistólica del ventrículo izquierdo, se inició el proceso de retiro de ECMO que se realizó a los 32 días de vida. Para ese momento, el paciente había permanecido en ECMO por 121 horas y 26 minutos.

El proceso de recuperación fue largo, pero favorable. La fracción de eyección de ventrículo izquierdo permaneció en torno al 70% desde el explante del ECMO. El paciente siguió a cargo del equipo multidisciplinario del servicio de Cardiopediatría hasta su alta a los 5 meses de vida.

Durante la atención del paciente se realizaron 18 diagnósticos de enfermería, de los cuales tres son propuestas que no están incluidas en la clasificación NANDA y se presentan en la Tabla 1. Además realizamos 53 intervenciones de las cuales 8 se incorporaron para dar continuidad al cuidado de enfermería, así como 24 clasificaciones de resultados de los cuales 4 fueron agregadas para dar continuidad en la evaluación de las intervenciones de enfermería realizadas. Asimismo, se continuó el proceso de atención de enfermería con diagnósticos e intervenciones según taxonomía NANDA II. (Tabla 2) Los principales cuidados fueron: identificación de riesgos, manejo de hipovolemia, regulación hemodinámica, valoración ácido base durante el ultra filtrado, precauciones de las urgencias en ECMO (vigilancia de embolismo, trombos, sangrado y desplazamiento de cánulas), aplicación de la lista de chequeo de reporte de enfermería y del funcionamiento adecuado del sistema, apoyo al cuidador principal y escucha activa.

**Tabla 1:** Proceso de atención de enfermería: Diagnósticos e intervenciones sugeridas para la continuidad en la atención del paciente neo-pediátrico en ECMO

Diagnósticos de Enfermería	Clasificación de Intervenciones de Enfermería (NIC)
Riesgo en las alteraciones de la entrega, demanda y consumo de oxígeno.* Factor de riesgo: Falla mecánica del equipo de ECMO y de la membrana oxigenatoria.**	Aplicación del checklist de reporte de enfermería del funcionamiento del equipo de ECMO.*** Verificación de la posición de cánulas (señalizar sobre la piel la fijación de las cánulas).*** Verificar el adecuado funcionamiento de la membrana (valoración de gasometría pre y post membrana),** terapia de ultrafiltración, balance hídrico estricto valoración de los resultados de TCA culminando el ultra filtrado, valoración ácido-base, durante el procedimiento de ultra filtrado.***
Riesgo de lesión (00035) (decanulación accidental, acodaduras, émbolos). Factor de riesgo: Transporte del paciente en ECMO.**	Precauciones de las urgencias en ECMO, vigilancia de embolismo, presencia de trombos y desplazamiento de cánulas.*** Aplicación de los checklist de reporte de enfermería del paciente en ECMO y de verificación del funcionamiento adecuado del sistema.*** Transporte dentro de la instalación (7892), aplicación del checklist de transporte del paciente en ECMO. ***
Riesgo de disminución en el flujo sanguíneo del paciente sometido a ECMO.* Factor relacionado: Incremento de la postcarga no detectado por la máquina de ECMO.**	Mantener una adecuada presión sistémica, uso adecuado de vasodilatadores e inotrópicos, valoración de informes de ecocardiografía, registro y comunicación de cambios hemodinámicos del paciente.***

*Diagnósticos de enfermería propuestos que no están incluidas en la clasificación NANDA.

** Factor de riesgo propuesto que no está incluido en la clasificación NANDA.

*** Intervenciones de enfermería propuestos que no están incluidas en la clasificación NIC.

**Tabla 2:** Proceso de atención de enfermería: Diagnósticos e intervenciones según taxonomía NANDA II

NANDA	NIC / NOC relacionado
Diagnóstico: Riesgo de disminución de la perfusión tisular cardíaca Dominio 4 Actividad / Reposo Clase 4 Respuestas cardiovasculares/pulmonares Código 00200 Edición: Aprobado 2008. Revisado 2013, 2017. Nivel de evidencia 2.1. Definición: Susceptible a una disminución de la circulación cardíaca (coronaria) que puede comprometer la salud. Factor relacionado: Taponamiento cardíaco, hipovolemia.	NIC Administración de medicación (2300), Colaboración con el médico (7710) Control de la medicación (2395) Manejo de la medicación (2380) Cuidados cardíacos (4040) Cuidados circulatorios: dispositivo de ayuda mecánico (4064) Monitorización hemodinámica invasiva (4210) NOC: Efectividad de la bomba cardíaca (0400) Estado circulatorio (0401) Estado cardiopulmonar (0414) Respuesta de la medicación (2301)
Diagnóstico: Riesgo de perfusión tisular cerebral ineficaz Dominio 4 Actividad / Reposo Clase 4 Respuestas cardiovasculares/pulmonares Código 00201 Edición: Aprobado 2008. Revisado 2013, 2017. Nivel de evidencia 2.1. Definición: La susceptibilidad a una reducción en la circulación del tejido cerebral que puede poner en peligro la salud. Condiciones asociadas: Régimen del tratamiento, anticoagulación propia de la terapia ECMO, tiempo de protrombina, tiempo de tromboplastina.	NIC Mejora de la perfusión cerebral (2550) NOC: Perfusión tisular cerebral (0406) Reflejos neurológicos alterados (040620) Valoración del NIRS
Diagnóstico: Riesgo de infección Dominio 11 Seguridad / Protección Clase 1 Infección Código 00004 Edición: Aprobado 1986. Revisado 2010, 2013, 2017. N. de evidencia 2.1. Definición: Susceptible a invasión y multiplicación de organismos patógenos que puede comprometer la salud. Factor de riesgo: Alteración en la integridad de la piel, defensas primarias insuficientes, defensas secundarias insuficientes. Condiciones asociadas: Procedimiento invasivo, respuesta inflamatoria suprimida.	NIC Control de infecciones (6540) NOC: Control de riesgo: proceso infeccioso (1924) Uso de medidas de bioseguridad durante el acceso a dispositivos invasivos (cánulas de salida y de retorno del paciente).
Diagnóstico: Riesgo de sangrado Dominio 11 Seguridad / Protección Clase 2 Lesión física Código 00206 Edición: Aprobado 2008. Revisado 2013, 2017. Nivel de evidencia 2.1. Definición: Susceptibilidad de reducción en el volumen de sangre que puede comprometer la salud. Factor relacionado: Régimen terapéutico (anticoagulación con heparina), taponamiento cardíaco, hipovolemia.	NIC Administración de hemoderivados (4030) Aspiración de las vías aéreas (3160) Cuidados del drenaje torácico (1872) Interpretación de datos de laboratorio (7690) Prevención de hemorragias (4010) NOC: Respuesta a la medicación (2301) Efectos terapéuticos (230101) Mantenimiento de los niveles sanguíneos esperados (TTP, TCA)

NIRS: Espectroscopía del infrarrojo cercano; TTP: tiempo parcial de tromboplastina; TCA: tiempo de coagulación activada.

**Figura 3:**
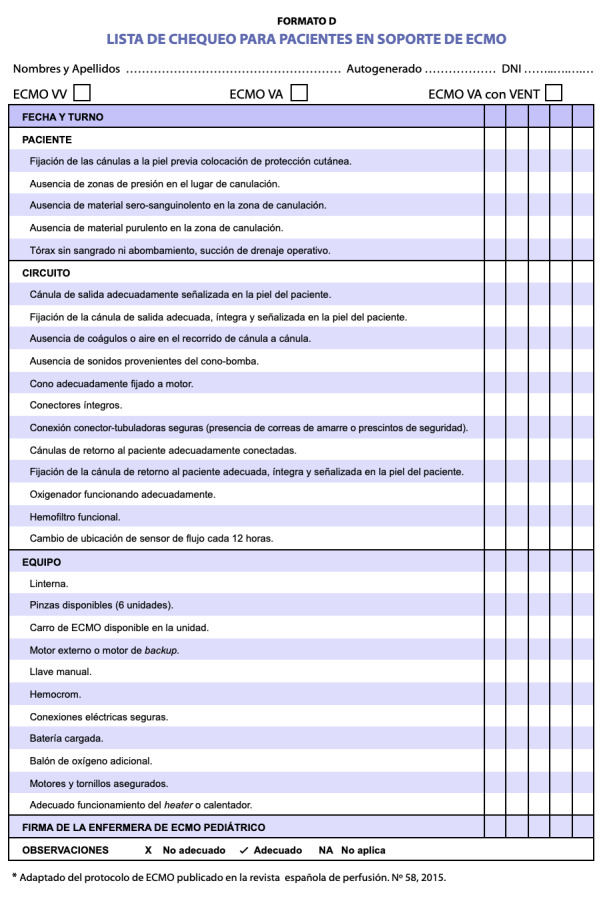
Lista de chequeo del paciente en soporte de ECMO.

Se adaptaron siete registros y listas de chequeo^2^ para el cuidado del paciente pediátrico sometido a dicha terapia: lista de chequeo para la preparación del paciente que será sometido a ECMO, lista de chequeo del reporte de enfermería del paciente pediátrico, lista de chequeo del traslado del paciente pediátrico, lista de chequeo del paciente en soporte de ECMO, registro de ECMO pediátrico, lista de chequeo para el paciente programado para ECMO del servicio de cardiopediatría y finalmente se elaboró la cartilla de urgencias del paciente neonato pediátrico en ECMO. (Figura 3)

## Discusión

En el Perú, este es el primer caso reportado de ECMO CENTRAL veno-arterial-VENT en neonatos. A nivel de Latinoamérica, existen reportes de casos de ECMO arterial en pacientes adultos; sin embargo, son pocos los trabajos de enfermería asociados al cuidado con las nomenclaturas NANDA,^3^ NIC^4^ y NOC,^5^ por lo que fue imperativo realizar los cuidados especializados bajo la experiencia y conocimientos teóricos del paciente neonato sometido a ECMO.

El caso implicó establecer un trabajo estandarizado y secuencial del cuidado de enfermería, el cual fue adaptado en base a los conocimientos obtenidos en capacitaciones realizadas en Brasil y Colombia. Este trabajo requirió cuidados de enfermería diferenciados de otro grupo asistencial.

En 2012, en el Complejo hospitalario Universitario de La Coruña - España, se reportó un caso clínico de ECMO en un paciente varón de 66 años donde se utilizó la taxonomía NANDA, NOC y NIC, evidenciando que al utilizar un plan de cuidados y evaluar los NOC la evolución fue favorable^.(^^6^

En 2015, en el Instituto del Corazón del Hospital de las Clínicas de la Universidad de Sao Paulo - Brasil, se pudo evidenciar la importancia de la sistematización de los cuidados de enfermería en una paciente mujer de 20 años de edad, que requirió ECMO tras trasplante pulmonar. Se establecieron 18 diagnósticos de enfermería y 94 conductas implementadas que fueron útiles en todas las fases del ECMO: implante, mantenimiento y explante del circuito.^7^

No hay casos reportados de neonatos en ECMO central veno-arterial con vent asociados al cuidado de enfermería con la taxonomía NANDA, NIC y NOC; siendo este, hasta nuestro entender, el primer caso publicado en Latinoamérica y en el mundo.

## Conclusión

La rigurosidad del reporte oral de enfermería es un pilar para garantizar la continuidad, secuencialidad, estandarización del cuidado de enfermería y aplicabilidad de las diferentes listas de chequeo. Es imperativo que la taxonomía NANDA, NIC y NOC contemple la incorporación de nuevos diagnósticos e intervenciones para el cuidado de pacientes en ECMO, que debe ser diferenciado para el grupo neonatal y pediátrico.

## Financiación:

Ninguna

## References

[1] Extracorporeal Life Support Organization ELSO (2014). GUÍAS DE LA ELSO PARA CENTROS ECMO. Version1.8.

[2] Rubia C, Reta L, Diez R (2015). Protocolo ECMO. Revista Española de Perfusión.

[3] (2019). Diagnósticos Enfermeros. Definiciones y Clasificación 2018-2020.

[4] Bulechek G, Butcher H (2014). Clasificación de Intervenciones de Enfermería (NIC).

[5] Sue Moretead, Marion Johnson (2014). Otra clasificación de resultados de enfermería (NOC).

[6] Moreno F, Pérez M, Poza A (2012). ECMO: un caso clínico. Enfermería en Cardiología.

[7] De Oliveira L, Neves A, Jardim J (2015). Uso de la membrana de oxigenación extracorpórea en una paciente con trasplante pulmonar cuidados de Enfermería. Enferm. Glob.

